# A randomized controlled TRIal of cognitive BEhavioral therapy for high Catastrophizing in patients undergoing lumbar fusion surgery: the TRIBECA study

**DOI:** 10.1186/s12891-020-03826-w

**Published:** 2020-12-04

**Authors:** P. Scarone, A. Y. J. M. Smeets, S. M. J. van Kuijk, H. van Santbrink, M. Peters, E. Koetsier

**Affiliations:** 1 Neurosurgical Service, Neurocenter of Southern Switzerland, Lugano, Switzerland; 2 Department of Neurosurgery, Zuyderland Medical Center, Heerlen/Sittard-Geleen, The Netherlands; 3 Department of Neurosurgery, Maastricht University Medical Center, Maastricht, The Netherlands; 4 School CAPHRI, Care and Public Health Research Institute, Maastricht University, Maastricht, The Netherlands; 5 Department of Clinical Epidemiology and Medical Technology Assessment, Maastricht University Medical Center, Maastricht, The Netherlands; 6 Department of Clinical Psychological Science, Maastricht University, Maastricht, The Netherlands; 7 Pain Management Center, Neurocenter of Southern Switzerland, Lugano, Switzerland; 8 Division of Anesthesiology, Department of Acute Medicine, Regional Hospital of Lugano, Lugano, Switzerland

**Keywords:** Lumbar spinal fusion surgery, Pedicle screws, Pain catastrophizing, Cognitive behavioral therapy (CBT), Education, Chronic postsurgical pain (CPSP)

## Abstract

**Background:**

Around 20% of patients undergoing spinal fusion surgery have persistent back or leg pain despite surgery. Pain catastrophizing is the strongest psychological predictor for chronic postsurgical pain. Psychological variables are modifiable and could be target for intervention. However, randomized controlled trials evaluating the effectiveness of psychological interventions to reduce chronic pain and disability after spinal fusion in a population of patients with high preoperative pain catastrophizing scores are missing. The aim of our study is to examine whether an intervention targeting pain catastrophizing mitigates the risk of chronic postsurgical pain and disability. Our primary hypothesis is that targeted perioperative cognitive behavioral therapy decreases the risk of chronic postsurgical pain and disability after spinal fusion surgery in high catastrophizing patients.

**Methods:**

We will perform a two-center prospective, single-blind, randomized, controlled study comparing lumbar spinal fusion surgery outcome between 2 cohorts. Adult patients selected for lumbar spinal fusion with decompression surgery and a minimum score of 24 on the pain catastrophizing scale will be randomized with 1:1 allocation for either perioperative cognitive behavioral therapy (intervention group) or a perioperative education plus progressive exercise program (control group). Patients randomized to the intervention group will receive six individual sessions of cognitive behavioral therapy, two sessions before the operation and four after. Primary outcome is the Core Outcome Measures Index at 12 months. Secondary outcomes include pain, disability, depression and quality of life.

**Discussion:**

This is the first trial that evaluates the effectiveness of cognitive behavioral therapy as a perioperative tool to improve pain and disability after spinal fusion surgery in comparison with an educational/exercise control intervention, in patients with high levels of pain catastrophizing. If perioperative cognitive behavioral therapy proves to be effective, this might have important clinical implications, reducing the incidence of chronic postsurgical pain and improving outcome after spinal fusion surgery.

**Trial registration:**

Clinicaltrials (NCT03969602). Registered 31 May 2019,

## Background

The incidence of moderate to severe chronic postsurgical pain (CPSP) 12 months after surgery is around 12% in Europe, depending in part upon the surgical procedure [1]. World-wide, more than 230 million people undergo major surgery every year, and the global annual costs for new cases of CPSP are estimated to be hundreds of billions of dollars [2]. Emphasis on identifying the processes that underlie the transition to chronicity has been a neglected topic of investigation [3]. Perioperative protocols have historically not incorporated routine screening for patients at risk for CPSP or compromised function. However, early detection and management of these patients may modify the postsurgical pain trajectory and reduce the incidence of CPSP. Several predictive factors for CPSP have been identified, the most important being chronic preoperative pain, high intensity of acute postoperative pain and several psychological factors [4]. Of these psychological factors, pain catastrophizing has emerged as a robust predictor of pain severity and work disability among individuals with persistent musculoskeletal pain and is the strongest predictor for CPSP [5–7]. Catastrophizing is considered as a maladaptive coping strategy involving an exaggerated response to anticipated or actual pain. Individuals who catastrophize tend to ruminate about pain, magnify the threat value of pain and feel helpless when dealing with pain [5]. It is even postulated that catastrophizing could have a direct influence on the neurophysiologic mechanisms involved in pain processing [8].

CPSP and disability after spinal surgery poses a large problem. Around 20% of patients have persistent or recurrent pain in the back or limbs despite spinal surgery [9, 10]. Pain catastrophizing is associated with pain and disability in patients with lumbar spinal stenosis [11], and patients undergoing spine surgery with high catastrophizing scores are more likely to have higher maximum pain scores in the immediate postoperative period [12, 13]. This can have long term consequences, as previous studies have shown that high levels of pre-operative pain catastrophizing are associated with higher levels of pain and disability at 6 and 12 months after spine surgery [14–16].

Psychological variables are modifiable and could be a target for intervention. Patients who have participated in psychological interventions for chronic pain, including cognitive behavioral therapy (CBT) and acceptance and commitment therapy (ACT), report less pain, pain-related disability, and mood disturbance [17, 18].

Although psychological treatments delivered in the perioperative period have the potential to influence the trajectory of CPSP in patients undergoing spinal fusion [19], surgical patients have historically not been offered these interventions. Additionally, it has been argued that treating ‘high catastrophizers’ with CBT can result in 50% fewer patients developing CPSP within this group [20]. In almost all centers that perform spinal surgery for degenerative conditions, patient selection is based on medical and psychiatric comorbidities [21, 22]. Even though the so-called “yellow flags” are well known by surgeons, very few attempts have been made to address these factors or to identify categories of patients that could be at risk for CPSP. Well-designed randomized controlled trials (RCT) to determine the effectiveness of a CBT approach to reduce chronic pain and disability after spinal fusion in a population of patients with high preoperative pain catastrophizing scores are needed.

The aim of our study is to examine whether a perioperative intervention that targets pain catastrophizing can mitigate the risk of CPSP, i.e. improve the outcome of the surgical intervention, in patients that report high levels of pain catastrophizing and for whom lumbar spinal fusion surgery is indicated. We will start CBT already before surgery, to give patients the appropriate tools to manage symptoms during the immediate postoperative phase. The control group is offered an educational plus progressive exercise program (hereafter labelled ‘educational program’). We hypothesize that perioperative CBT, specifically designed to reduce pain catastrophizing, will improve outcome and decrease the risk of CPSP and disability after spinal fusion surgery, assessed by the Core Outcome Measures Index (COMI) questionnaire in high catastrophizing patients. Additionally, we will include a cohort of low catastrophizing patients receiving usual care to compare the outcome of spinal fusion surgery in low catastrophizing patients with high catastrophizing patients after perioperative CBT.

## Methods/design

### Funding

This study has received funding from the Advisory Board of Ente Ospedaliero Cantonale (ABREOC) (NSI-TD/NCH-01), and from the Neurosurgical/Orthopedic research fund Zuyd of the Zuyderland Medical Centre Heerlen/Sittard-Geleen, the Netherlands.

### Study design

**Fig. 1:**
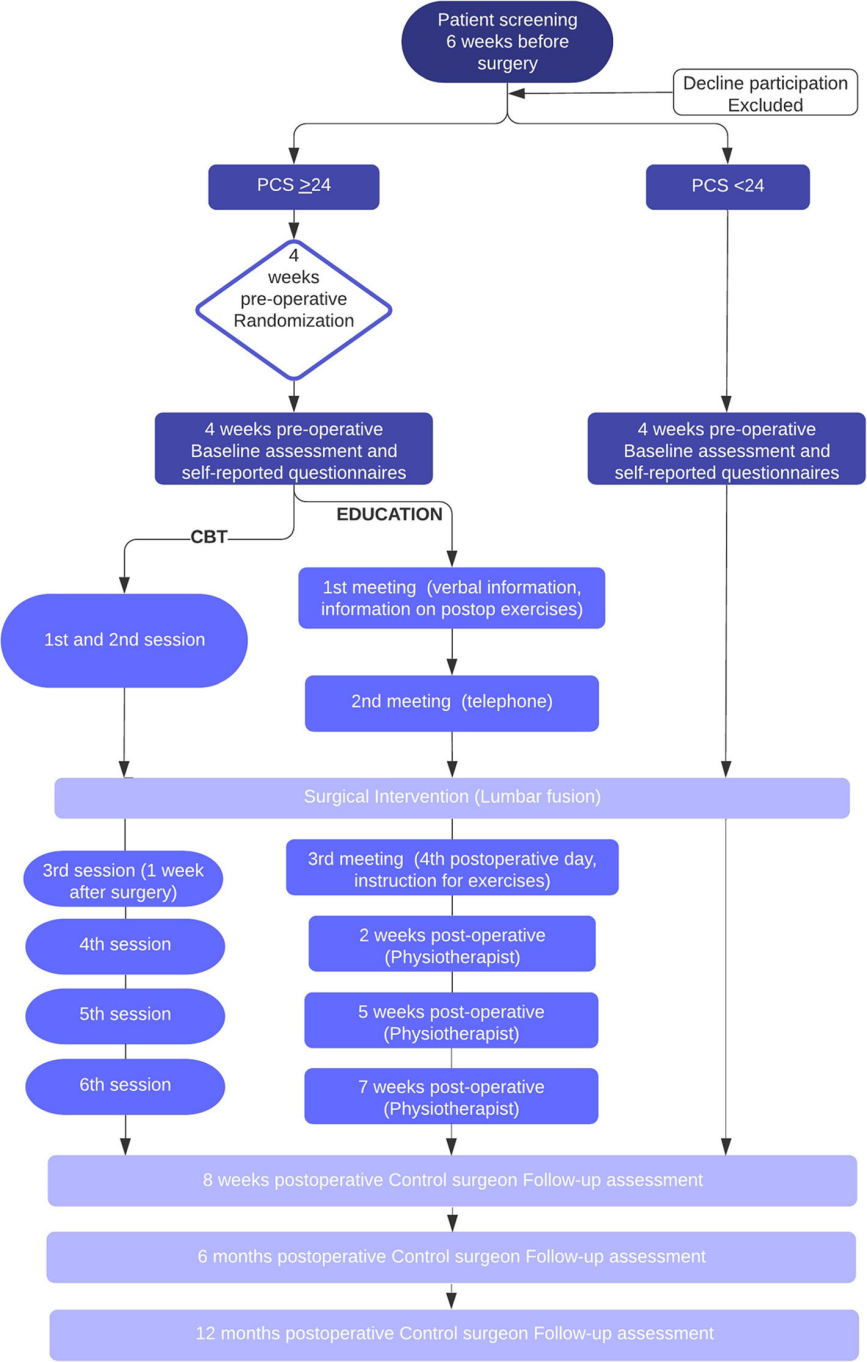
Study design, timing of pre- and postoperative sessions and follow-up evaluations

This is a two-center prospective, single-blinded, randomized, controlled trial, in high pain catastrophizing patients with an indication for lumbar spinal fusion and decompression surgery, comparing two groups: one that receives perioperative CBT and one that receives the educational program. Figure 1 illustrates the study design, timing of pre- and postoperative sessions and follow-up evaluations. Estimated duration for the main investigational plan (e.g. from start of screening of first participant to last participant processed and finishing the study) is 4 years.

### Ethical principles

Ethical approval has been granted by the Research Ethics Committee of the Canton Ticino, Switzerland (2018–00880 - CE 3361) and the Medical Ethical Committee Zuyderland, Heerlen, the Netherlands. Informed consent will be obtained in writing from all participants prior to study enrollment.

### Participants and recruitment

Adult patients who are undergoing surgery for a degenerative condition of the lumbar spine (lumbar spinal stenosis, spondylolisthesis or degenerative disc disease) using any kind of surgical decompression (laminectomy, hemilaminectomy, foraminotomy, with open or minimally invasive technique) associated with fusion, and able to read and speak Italian (site in southern Switzerland) or Dutch (site in the Netherlands) will be eligible for inclusion. One hundred and fifty patients will be enrolled at the outpatient clinic of the Department of Neurosurgery, Neurocenter of Southern Switzerland, Regional Hospital of Lugano, Lugano, Switzerland (Site 1) and the Department of Neurosurgery of Zuyderland Medical Center, Heerlen, The Netherlands (Site 2). All patients entering the screening phase will be registered on a Patient Registration Log and a unique registration number will be assigned. Clinical data will be assessed to evaluate a subject’s eligibility.

Patients will be excluded from the study if they meet any of the following criteria:

- Plans to undergo major surgery within six months after current lumbar spinal fusion surgery
- Comorbid severe psychiatric conditions such as schizophrenia or personality disorder
- Known or suspected non-compliance, drug or alcohol abuse
- Inability to follow the procedures of the study, e.g. due to dementia
- The presence of any serious medical comorbidity such as sepsis or cancer that might cause disability or worsen the patient’s general health condition
- Pregnancy
- An opioid intrathecal pump
- Prisoners

Patients eligible for participation will be evaluated at least 6 weeks before surgery and asked for informed consent, and then to complete the PCS [23]. Patients with high pain catastrophizing, defined as a score of ≥24 on the PCS, will be eligible for randomization to perioperative treatment with CBT or educational program. This cut-off was initially based on a previous study in whiplash patients that showed that a pretreatment score of 24 or higher on the PCS best predicted follow-up pain ratings and work status after multidisciplinary treatment [24]. More recently, Tuna et al. [16] suggested an almost identical cut-off for the PCS (i.e. > 24) to obtain the highest sensitivity and specificity to predict unfavorable outcome after spine surgery, defined by as score on the Oswestry Disability Index (ODI) of 20 or higher.

A non-randomized group, consisting of low catastrophizing patients (score < 24 PCS), will be included as an observational cohort. These patients will not undergo any additional intervention besides usual care but will undergo the same data-collection as the high catastrophizing randomized groups.

### Randomization

A computer-generated randomization will be used to allocate the participant to either CBT or educational program with a 1:1 allocation. We will use block randomization stratified by center, with block sizes of 6. The randomization is performed by the Principal Investigator per site, who is not blinded for group assignment. The patients will be randomized immediately after they are included and have completed the PCS (one of the baseline assessments) to be able to make two pre-operative appointments with the patients in the intervention and control group.

### Comparators

#### Cognitive behavioral therapy

The CBT protocol was specifically developed for the present study by a team of psychologists with longstanding experience in the treatment of chronic pain patients. The intervention consists of six sessions, individually delivered by clinical video teleconferencing, and specifically aiming to reduce pain catastrophizing [25]. Two sessions are delivered before surgery (resp. at 3–6 weeks and 1–3 weeks pre-surgery) and four sessions after surgery. The four post-surgical session will start approximately 2 weeks after surgery, with sessions spaced one week apart (allowing a range of 2–3 weeks to prevent scheduling difficulties). All sessions have a duration of 60 min except for the first session that will take 90 min. Details of timing and the content of each sessions can be found in Table 1. In short, sessions focus on pain education, identifying, challenging and replacing catastrophizing thoughts, discussing fears of surgery, activities and goals after surgery, consequences of avoidance behavior, adaptive means for accomplishing interpersonal needs and relapse prevention. It also includes progressive muscle relaxation (PMR) training to provide patients with tools to cope with acute postoperative symptoms and to enhance their feelings of control. The protocol is manualized and the therapists delivering the treatment record after each session which components have been addressed. Patients receive a workbook consisting of a brief summary of the pain education, and for each session an explanation of the exercises for that week, several pages with forms to fill in and some examples. The homework is sent to the therapist before the start of the next session. Patients also receive an audio file with a recording of the PMR exercise that they are requested to practice at least once a day.

**Table 1:** timing and content of cognitive behavioral therapy sessions

Timing	Aim	Content
Session 1 6–3 weeks pre-surgery 90 min	Building relationship Providing treatment rational Pain education Teaching relaxation	Gathering personal, medical and pain history Education on association between, cognitions, emotions, behavior and consequences Education on disconnection of “damage” or injury and perceived pain Building awareness of the impact of stress and fear on pain Explaining goals of the six sessions Practicing progressive muscle relaxation *Homework*: ABC belief monitoring (registering thoughts, emotions and consequences in pain situations) List “difficult” situations Practice progressive muscle relaxation
Session 2 3–1 weeks pre-surgery 60 min	Identifying catastrophizing thoughts Discussing fears of surgery	Homework review from previous session Introduction of the concept “catastrophizing” Identifying catastrophizing thoughts from homework Practicing with challenging and replacing catastrophizing and other maladaptive thoughts *Homework*: ABC belief monitoring (diary) Practice relaxation techniques
Session 3 1–3 weeks post-surgery 60 min	Challenging and replacing catastrophizing thoughts	Homework review Identify and replace catastrophizing thoughts Discussion of new fears and challenges *Homework*: ABC belief monitoring (diary) Practice relaxation techniques
Session 4 2–5 weeks post-surgery 60 min	Challenging and replacing catastrophizing thoughts Overcoming avoidance Learning new ways of coping	Homework review Discuss to which maladaptive behavior catastrophizing might lead Discuss how to attain goals in an alternative way Identify barriers for adaptive coping Discuss how to deal with upcoming stressful situations *Homework*: ABC belief monitoring (diary) Identify needs being met by avoidance behavior and list alternative options for attaining those needs Practice relaxation techniques
Session 5 3–6 weeks post-surgery 60 min	Learning more adaptive means for accomplishing interpersonal needs	Homework review Note and discuss progress Continue with challenging and replacing catastrophizing thoughts Discuss challenges from previous week Focus on how to communicate needs to environment and identify barriers in doing so. *Homework*: ABC belief monitoring (diary) Practice relaxation techniques
Session 6 4–8 weeks post-surgery 60 min	Maintenance Promoting continued practice	Discussing challenging catastrophizing thoughts as a longterm project Reviewing and summarizing the most important concepts Patients receive the completed homework binder

Each patient will be treated by a single therapist. At the Swiss site, CBT will be delivered by one of the clinical psychologists who was involved in developing the protocol. At the Dutch site, CBT will be delivered by one of two clinical psychologists. Both have prior experience in pain management and are additionally trained in working with the current protocol by one of the psychologists involved in its development. All CBT providers have to follow the guideline that was created for this study, to assure that all sessions will be held in the same way. All patients in the CBT group will receive a workbook with pages to fill out for every session and with some additional exercises.

#### Educational program plus progressive exercise program

The control intervention consists of biomedical and surgery-specific education before surgery and an exercise manual after surgery. To control for differences in attention between the two interventions, patients in the educational program will also have two meetings before and four meetings after surgery with members of the research team (in person or through telephone).

The pre-surgical, biomedical and surgery-specific education is based on a previously developed educational intervention for spinal patients [26]. In the first preoperative in person meeting (30–45 min), a ‘not-blinded’ neurosurgeon will provide verbal information on the preparation for surgery, surgery itself and recovery from surgery, stressing the importance of postoperative exercises. Questions raised by the patient are discussed. A booklet is provided with information about the following topics: structure of the spinal column and spinal diseases; examinations before surgery; the operative environment; surgical procedures; anesthetic procedures; postoperative care and postoperative pain reduction. A second telephone meeting a second telephone meeting of 10–15 min with the same neurosurgeon is planned 1–2 weeks after to answer question about the given information and to discuss point that are unclear.

After surgery and before discharge from the hospital, a physiotherapist will meet the patient (meeting of circa 20 min) and provide him with instructions about the exercise program. Patients will receive a manual describing exercises to be performed in the weeks following surgery. The exercise program is based on the program of Abbott et al. [27] and consist of dynamic exercises to gradually enhance endurance capacity of the back and leg muscles, stretching and cardiovascular exercises. Patients keep a daily diary, for the duration of 8 weeks, tracking on paper their training activity, signing a ‘X’ every time they performed their daily muscle reinforcement exercises, stretching exercises and cardiovascular exercises. Two, five and seven weeks after discharge patients are contacted by telephone (a circa 20 min telephone meeting) by a physiotherapist. For safety reasons, patients will be asked to increase the number of repetitions as long as their pain remains tolerable. They will be asked to gradually increase the repetitions according to their perceived pain. During the telephone sessions, their training progress is discussed and patients are remembered to mark their training activity in the diary. Patients are asked to bring the diary to the 2 months follow-up visit, to check for compliance to the training instructions. The providers of the educational sessions will be trained and have to follow the manual that was created for this program, to assure that all sessions will be held in the same way. Although not exactly the same, the time points of the fourth, fifth and sixth educational session come close to the time points of the fourth, fifth and sixth session of the CBT intervention, which are at 2–5 weeks, 3–6 and 4–8 weeks post-surgery, respectively. The providers of the educational sessions will be trained and have to follow the manual that was created for this program, to assure that all sessions will be held in the same way in both sites.

All patients, independently from randomization into one of the groups, will be routinely prescribed medications for pain control and will be referred for physical therapy on an out-patient basis in different sites or an in-patient basis in specialized rehabilitation centers following lumbar spinal fusion surgery, depending on the needs.

### Outcome measures

Primary and secondary outcomes will be measured at baseline preoperatively and 2, 6 and 12 months after surgery (Table 2). Investigators and research assistants collecting outcomes from patients at different time points during follow-up will be blinded to group assignment. The psychologists delivering the CBT and the clinicians providing the educational program to the control group cannot be blinded. Nevertheless, patients will be blinded to the study hypotheses and will be informed that the study is examining the comparative benefits of two treatments that are given in addition to usual care and that may potentially improve outcomes. The patients will not be informed about the likely association between a high PCS score and lower outcomes after spinal fusion surgery, neither about the fact that the control intervention will not address catastrophizing.

**Table 2:** Outcome measures. (Days-D, Weeks-W, Months-M)

***Outcomes***	***Instrument***	***Study Period***	***Enrollment***		***Surgery***					
		***Time***	***≥ 6 W***	***Intervention*** ***Between 4 W preop***	***Baseline***	***4D***	***Intervention*** ***Between 8 W postop***	***8 W***	***6 M***	***12 M***
*Primary outcome:*										
Self-reported function	COMI		X					X	X	X
*Secondary outcomes:*										
Self-reported Pain intensity	NRS		X			X		X	X	X
Self-reported disability	ODI		X					X	X	X
Self-reported daily-function and quality of life	EQ-5D		X					X	X	X
Self-reported mood and cognition	PROMIS depression		X					X	X	X
Patient’s global impression of change	PGIC		X					X	X	X
Self-reported pain catastrophizing	PCS		X					X	X	X
Medication Usage	MQS		X					X	X	X
Work productivity and activity impairment	WPAI:LBP		X					X	X	X
*Other variables:*										
Patient demographics	Medical record		X							
Surgical complications	Clavien-Dindo							X		
Self-reported anxiety	PROMIS anxiety		X					X	X	X
Self-reported fear of surgery	SFQ		X					X	X	X
Self-reported fear avoidance beliefs	FABQpa		X					X	X	X

#### Primary outcome

The primary outcome is the patient’s score on the COMI 12 months after surgery. The COMI is a self-administered multidimensional instrument that consists of seven items which assess the extent of the patient’s back pain and leg pain, difficulties with functioning in everyday life, symptom-specific well-being, general quality of life, and social and work disability. Leg pain and back pain are assessed on 0–10 graphic rating scales and all other items on 5-point adjective scales. In each case, a higher score indicates worse status [28]. The instrument is validated in Italian and Dutch [29, 30].

#### Secondary outcomes

Secondary outcomes are leg/back pain, disability, quality of life, depression, patient’s global impression of change, pain catastrophizing, pain medication regimen, and work productivity and activity impairment. These will be measured using the following questionnaires:

- Numeric Rating Scale (NRS) for back and leg pain. Pain intensity is assessed on an 11-point scale ranging from 0 (no pain at all) to 10 (the worst imaginable pain) [31].
- Oswestry Disability Index (ODI): a self-administered questionnaire, assessing a patient’s limitations in activities of daily living. The ODI has ten questions scored on a 6-point scale. The total score ranges from 0 to 100, with higher scores denoting higher functional disability [32].
- European Quality of Life Five Dimension Five Level Scale (EQ-5D-5L): This questionnaire assesses health related quality of life in terms of five dimensions: mobility, self-care, usual activities, pain/discomfort, and anxiety/depression [33]. Each of the 5 items are scored on a 5-point scale, ranging from no problems in that domain to extremely problematic.
- Depression is measured by the PROMIS (Patient-Reported Outcomes Measurement Information System) Short Form v1.0 - Depression 8b [34]. The eight questions are scored from 0 to 5. Higher scores denote higher levels of depression.
- Patient’s Global Impression of Change (PGIC): This scale assesses patient’s own assessment improvement or deterioration over time on a 7-point scale (1. very much improved - 7. very much worse).
- Pain Catastrophizing Scale (PCS): The PCS is a 13-item scale that measures catastrophic thinking in relation to pain. Each item is scored on a 5-point scale ranging from 0 (never) to 4 (always) [23]. Higher scores denote higher levels of catastrophizing. Psychometric properties of the PCS have been studied extensively in a variety of patient populations having painful disorders and found to be good to excellent [35, 36]. In addition, the PCS has been shown to be sensitive to psychosocial interventions designed to increase the use of adaptive coping strategies and decrease the use of maladaptive coping strategies, such as pain catastrophizing [37].
- Medication Quantification Scale (MQS): The MQS is an instrument or quantifying medication regimen use in chronic pain populations. The score is calculated for each medication by taking a consensus-based detriment weight for a given pharmacologic class and multiplying it by a score for dosage. The calculated values for each medication are then summed for a total MQS score. The score can provide a useful point measure of medication usage for any pain medication regimen [38, 39].
- Work productivity and activity impairment will be assessed using the Work Productivity and Activity Impairment questionnaire: Low Back Pain (WPAI:LBP) [40]. This questionnaire measures the effect of the specific health problem ‘low back pain’ on work productivity and activity impairment. Specific outcomes are absenteeism (work time missed), presenteeism (impairment while working), overall work impairment (absenteeism plus presenteeism), and activity impairment (impairment in regular activities) due to LBP. Each score is represented as a percentage, and higher scores indicate less productivity or greater impairment.

#### Other variables of interest

The following patient characteristics will be gathered: gender, age, weight, height, civil state, employment status (e.g. retired, no paid work), type of working activity (e.g. sedentary, physical, job absent or reduced due to the pain), educational level, smoking status, allergies, low back pain duration, other chronic pain complaints, sickness benefits and length of current sick leave, self-reported psychiatric problems, and concomitant physiotherapy.

The following medical data will be gathered: type of operation, surgical indication, American Society of Anesthesiologists (ASA) classification.

Preoperative state anxiety will be assessed by the PROMIS (*Patient-Reported Outcomes Measurement Information System*) Short Form v1.0 – Anxiety 4a. The PROMIS Anxiety instruments measure self-reported fear (fearfulness, panic), anxious misery (worry, dread), hyperarousal (tension, nervousness, restlessness), and somatic symptoms related to arousal (racing heart, dizziness). Additionally, surgical fear will be measured by the ten-item Surgical Fear Questionnaire as a predictor of physical and emotional recovery [41]. Each item refers to a specific potential consequence of the operation (intervention) and is scored on an 11-point scale (from no fear to most extreme fear).

Fear avoidance belief will be assessed with the four Fear Avoidance Beliefs Questions that make up the “physical activity” part of the FABQ (denominated FABQpa) [42]. This is a patient reported questionnaire that specifically focuses on how a patient’s fear avoidance beliefs about physical activity and work may affect and contribute to their low back pain and resulting disability. Moreover, surgical complications will be recorded using the Clavien-Dindo classification system for postoperative complications of spinal fusion (therapy-oriented, 4-level severity grading) [43]. There will be no reporting of (severe) adverse events as this study is investigating the effect of a therapy (CBT therapy versus education/exercises) which does not pose a specific risk to the patients. At most patients might experience a mild emotional discomfort, but this is very seldom as these therapies are known to give benefit to patients.

### Power considerations and statistical analysis

The primary outcome is the difference in the COMI between the study groups, 12 months after surgery. A difference of two points is considered clinically meaningful [44]. With an assumed SD of 2.5, the effect size of this difference, quantified as Cohen’s *d* is 0.80. Inclusion of 42 patients per intervention group will yield a power of 0.95 at a type-I error rate of 0.05. In addition, 42 patients with low pain catastrophizing scores will undergo the same assessments, but not receive any intervention besides care as usual. Taking into account a drop-out rate of 15%, 150 patients will be included in total.

Baseline patient characteristics will be stratified by group and presented as mean and standard deviation (SD), median and first and third quartile, and count and percentage, as appropriate. Differences in baseline characteristics between randomized groups will not be statistically tested, whereas differences with the separate control cohort will be tested using the independent-samples t-test for continuous variables, and Pearson’s chi-square test for categorical variables. In case of expected cell counts of less than 5, Fisher’s Exact test will be used instead.

All patients randomized (i.e. those with a PCS score > 24) will be analyzed in an intention-to-treat analysis. To test for a difference between the CBT and control group in the COMI at 12 months, linear regression will be used, with correction for baseline COMI and center. In addition, the difference in the trajectory of COMI over time will be assessed by means of linear mixed-effects regression, taking all follow-up measurements into account. Adjustment for center, type of surgery, age and gender will be made and, if necessary, for other prognostic variables significantly associated with the outcome (i.e., acute postoperative pain intensity, severity of catastrophizing, preoperative depression, preoperative surgical fear, length of current sick leave, pain duration). Stepwise backward elimination using the Wald test will be used to select prognostic variables significantly associated with the outcome. Any control variables that are incomplete will be imputed if the proportion of incomplete patients exceeds 0.05. We will use multiple imputation with fully conditional specification with the number of imputations set to the percentage of incomplete patients. Predictive mean matching will be used to draw values to be imputed. Secondary outcome variables will be analyzed similar to the primary outcome. For these, logistic regression analysis adjusted for center will be used to test for differences in proportions at 12 months postoperatively.

Exploratory, we will compare the outcomes of the patients in the two intervention groups with the outcomes of patients with low pain catastrophizing scores (i.e. those with a PCS score < 24) undergoing care as usual. Linear mixed-effects regression for the three groups will be performed with primary and secondary outcomes at the three postoperative assessment periods and controlling for preoperative values. This analysis will indicate whether high catastrophizing patients follow a similar trajectory of pain and functioning after spinal fusion surgery compared non-catastrophizing patients.

## Discussion

Over the past 15 years, an increase in the number of spinal fusion procedures for degenerative diseases has been observed. A recent analysis on a large national database [45] reported a 62% increase in volume of these procedures from 2004 to 2015. This could reflect an aging population, but also an improvement in the diagnostic process, intraoperative technologies (like spinal navigation) and perioperative care of these patients. However, while a clear benefit in terms of pain, disability and return to work has been shown in patients suffering from spinal instability [46–48], recent RCT’s [47, 49] have questioned the usefulness of spinal fusion procedures for other conditions like spinal stenosis or degenerative spondylolisthesis. As a matter of fact, in many situations and in many health systems, decision-making regarding indications for spinal fusion is driven by surgeons’ and patients’ preferences, hospital factors and reimbursement policies [45, 50].

Controversies about indications for spinal fusion pose specific problems since these patients are at risk for CPSP, which has a significant impact on patient’s quality of life and can even lead to new-onset psychiatric diseases [51]. The biopsychosocial model views pain as the result of the dynamic interaction between psychological (mood, personality, behavior, cognition etc.), social (cultural, familial, socioeconomic, medical, etc.) and biological (genetic, biochemical, etc.) factors [52]. Therefore, treatment should not only focus on the biological aspects of pain (e.g. the spinal pathology and surgery), but also take into account psychosocial aspects. As especially pain catastrophizing has emerged as a robust predictor of CPSP, interventions aimed at reducing this risk factor may improve treatment outcome. CBT is the most frequently used psychological intervention for patients with chronic pain and a recent meta-analysis indicated that it is the most effective treatment for decreasing pain catastrophizing in targeted populations [53].

So far, only a few studies have investigated the impact of a psychological intervention in the perioperative period in patients undergoing spinal fusion [54–57]. Monticone et al. [54] evaluated the effect of CBT for the management of catastrophizing after lumbar fusion surgery. Patients were randomly assigned to exercise plus CBT (*n* = 65) or a control group, consisting of exercises only (n = 65). Exercise plus CBT was found to be superior to the ‘exercise only’ program in reducing disability, dysfunctional thoughts, and pain, and enhancing the quality of life, with effects lasting for at least 1 year after the end of intervention. Abbott et al. [27] offered psychomotor therapy focusing on cognition, behavior, and motor relearning to 53 patients after lumbar fusion surgery and compared outcomes in terms of pain, disability and quality of life to outcomes in 54 patients receiving ‘exercise only’. The psychomotor group reported less disability and higher quality of life until 12 months after the intervention and less pain until 6 months. An important limitation of both studies [27, 54] was that an active control intervention offering more than exercises alone was not included. As exercises were also part of the experimental condition and could be interpreted as ‘usual care’, it cannot be excluded that non-specific factors such as contact time and attention might have played a role. Moreover, treatment expectations might have differed between conditions.

Two more recent studies offered both pre- and postoperative CBT sessions to spinal fusion patients. Lotzke et al. [58] examined the effects of a ﻿person-centred physical therapy rehabilitation program based on a cognitive-behavioral approach consisting of four preoperative and one postoperative session. Fifty-nine patients were randomized to the program and 59 received care as usual. Both groups showed a significant improvement in pain and disability at 6 months’ follow-up, with no differences between the groups. Rolving et al. [55] randomized patients to CBT plus physiotherapy (*n* = 59) or physiotherapy only (*n* = 31). The intervention consisted of 4 preoperative and 2 postoperative CBT sessions. At 1-year follow-up, no significant difference in disability was found between the two groups, but the CBT group achieved a significant reduction in disability earlier than the control group.

It should be noted that none of the above studies preselected patients based on a psychological high-risk profile (i.e. high pain catastrophizing or anxiety/fear) despite the fact that at least some of these interventions were specifically designed to reduce these risk factors. Lotzke et al. speculated that their null finding may have been due to the high degree of variability in fear-avoidance beliefs in their patients [59]. Therefore, they recommended that future studies should evaluate the effects of psychological treatment specifically in patients with a high psychological risk profile for poor outcome.

In line with this recommendation, a crucial aspect of the current study is that we will offer CBT specifically to patients reporting high levels of pain catastrophizing. Another feature, which sets out study protocol apart from most previous studies in this area, is the inclusion of an active control intervention consisting of an educational and progressive exercising program. This allows us to assess the effect of CBT per se and control for non-specific effects such as expectancy, contact time and attention. In addition, starting treatment before surgery may be expected to lead to more profound intervention effects than post-surgical intervention only, because it might decrease early postoperative pain, which is among the most prominent predictive factors for developing CPSP [60]. Finally, we aim to assess the meaningfulness and clinical usefulness of the perioperative CBT intervention by comparing the outcomes of the high PCS/CBT group not only with the control group, but also to the outcomes of the low risk group (i.e. PCS score < 24). This offers the possibility to examine whether CBT can reduce the negative effects of catastrophizing on outcome to the level of improvement one would expect in non-catastrophizing patients.

There are some limitations of our study protocol that should be mentioned. The main limitation is patient’s compliance to the intervention. In fact, very few data exist that analyze the impact and feasibility of CBT in the perioperative period after spinal fusion surgery. Another possible limitation is related to potential differences in levels of catastrophizing between the populations of the two centers. Moreover, the exact proportion of high catastrophizing patients (defined in this study as having a PCS ≥ 24) is not known in advance and could vary between the two centers. Additionally, a potential weakness of our study protocol is related to the control condition, conceived to reduce patient’s expectation bias, augmenting contact time and attention, and therefore reducing non-specific effects on outcome. It is well known in psychological research that expectation is one of the most important non-specific factors that may modulate patients outcomes [61]. However, it is difficult to know if this intervention will control for other non-specific factors that could be present (e.g. patient motivation or the therapeutic relationship between the psychologist and patients during CBT). Additionally, because of the difference in length of sessions between the CBT sessions and the education program (the latter being of a shorter duration) the factor of ‘contact time’ is not perfectly controlled for. On the other hand, the first educational session and post-discharge educational session will be held ‘face to face’ whereas all CBT sessions will be delivered by video teleconference. This ‘in-person attention’ in the two educational sessions might compensate for the shorter contact time in the control group in comparison to the CBT group.

Furthermore, this study can demonstrate efficacy of the CBT intervention for high catastrophizing patients, but it does not assess cost-effectiveness. Although some cost elements are included (medication use, further treatment, employment status), no full cost analyses will be conducted. Moreover, if this study shows a positive effect on pain and disability from perioperative CBT, this will not clarify the optimal dosage of the intervention or the ideal timing. Finally, it cannot clarify whether a PCS score of 24 or higher is the most optimal cut-off for offering patients CBT. Future studies should conduct sensitivity analyses to establish at which level of pain catastrophizing intervention should be offered, to come to an optimal cost-benefit ratio.

In conclusion, the results of this study are expected to provide valuable information that will potentially change clinical practice of lumbar spinal fusion surgery. If our study can demonstrate that perioperative CBT is effective in improving clinical outcome after lumbar spine fusion surgery in high catastrophizing patients, it is highly recommended to perform prescreening to identify this subgroup for which a perioperative psychological treatment will be indicated. If CBT will not prove to be superior and if this study will confirm that high levels of pain catastrophizing have a negative impact on clinical outcome, that might be a reason to be very reluctant in offering these patients a spinal fusion procedure, trying to opt for less invasive treatment options.

## Trial status

Participant recruitment started in February 2020 and is planned to continue until November 2022. An interim analysis is planned after 6 months follow up of 21 patients. It is expected that data regarding the intervention effects will be available at the end of 2023.

## Data Availability

No data has been collected yet. All methods/questionnaires have been described in the study protocol.

## Abbreviations

ACT: Acceptance and Commitment Therapy
ASA: American Society of Anesthesiologists
CBT: Cognitive Behavioral Therapy
CVT: Clinical Video Teleconferencing
COMI: Core Outcome Measures Index
CPSP: Chronic postsurgical pain
DDD: Degenerative disc disease
EQ-5D: EuroQualityOfLife-5 dimensions
FABQpa: Fear Avoidance Beliefs Questionnaire
LBP: Low back pain
MQS: Medication Quantification Scale
NRS: Numeric Rating Scale
ODI: Oswestry Disability Index
PCS: Pain Catastrophizing Scale
PGIC: Patient’s global impression of change
PROMIS: Patient-Reported Outcomes Measurement Information System
PMR: Progressive muscle relaxation
PSQ: Pain Sensitivity Questionnaire
RCT: Randomized controlled trial
SD: Standard Deviation
SFQ: Surgical Fear Questionnaire
WPAI:LBP: Work productivity and activity impairment: low back pain
